# Wisconsin State Medical Society

**Published:** 1869-01-15

**Authors:** 


### PROCEEDINGS OF SOCIETIES.

Our thanks are due to the secretary, H. P. Strong, M. D., for the manuscript advance copy of the proceedings of the

### WISCONSIN STATE MEDICAL SOCIETY.

The society convened at Madison, June 10, 1868, Dr. H. Van Dusan, president, in the chair, and Dr. A. J. Ward, secretary, *pro tem.* After formal business, reception of new members, etc., discussion occurred with reference to issuance of diplomas by the society; with regard to examinations for life insurance; services as experts in the courts, etc., etc.

Dr. Cody reported a case of fracture of the ribs, the interesting point of the case being the emphysema that ensued, it having extended over the whole body. There was also a large amount of hæmorrhage of the lung.

Dr. Whiting spoke of the danger of fracture of the ribs, and did not believe that in half the cases reported there was any fracture.

Dr. Taggart thought the danger depended upon displacement of the bone.

Dr. Barrett said he had known of ten or twelve cases that had been reported in his town, and he did not know of but two of the cases that were really fractured.

Dr. Brown offered the following resolution, which was adopted:

*Resolved,* That in explanation of the action of the society heretofore, upon the subject of admission to membership, the following conditions are required: The applicant shall present to the Board of Censors a diploma from a regular organized school of medicine of good repute, or a certificate or diploma of membership in a county or district society, accompanied with a certficate of six years practice, and of good moral and professional character, or he shall submit to such an examination as the Censors may impose.

Dr. Ward asked leave to present a patient to the society for opinion as to the nature of the disease, which was granted, and an examination made by some of the members of the association present. The patient was a lady of about 30 years of age. After confinement, sometime since, she left her bed rather too early, and was attacked with roaring in the right side of the head. This is constant and very annoying. Instant relief is obtained by pressure upon the common carotid artery, and remains so long as the pressure continues. This is the only means by which relief is afforded. There was a diversity of opinion as to the nature of the difficulty, and no definite conclusion obtained.

Dr. Marks, chairman of committee on surgery, made a report, at length, on treatment of fractures of the thigh. The report goes on to prove, by the best authority, that oblique fractures of the femur in adults nearly always results in more or less shortening. He also proved that the majority of surgeons prefer the straight position in treatment of these fractures, and goes on to say that, "where there is displacement of the fragments, the points of bone must be in contact with the soft parts, and that any unnecessary handling or twisting of the limb inflicts injury upon the muscles and other tissues, which taxes nature to repair. Gentleness is not incompatible with good surgery, although there are men who, judging from their method of manipulation, would differ with me. Those claiming to be good surgeons often inflict an amount of unnecessary injury to the soft parts, in their examinations, that takes nature weeks, I might say months, to repair." The treatment he has adopted and prefers, is to use extension by means of adhesive plaster applied to the leg, terminating below the foot, to which is attached a weight running over a pulley, varying in size from eight to twento pounds in adult cases. A sand bag is placed on each side of the limb, and the foot of the bedstead elevated three or four inches, the weight of the body being the counter extension. When the fracture occurs below the lesser trochanter he applies pasteboard splints the whole length of the limb upon either side, using the extension as before indicated.

The advantages claimed for this method are: First, ease and comfort to the patient. Second, the dressing does not interfere in the least with the circulation in the limb. Thirdly, the limb can be seen at all times, and in case of compound fracture the wound can be dressed as often as desirable. Fourthly, the limb can be dressed and placed in position in one-fourth the time required to apply the splint and roller. Fifthly, "I claim better results from this method than from any other I have ever tried, though I don't pretend that it will always prevent shortening."

In fractures within the capsule, or partly within, he uses only sand-bags with the weight and pulley, as before alluded to. Dr. Marks gave a history of several cases treated in this manner, and the results warrant a general trial.

Dr. Mason, of Prairie du Chien, was called upon to give the result of his experience in the treatment of fractures without splints. He made a statement that he had treated fractures of the femur and tibia by the use of sand-bags and the weight with good success, and had also used the same treatment for synovitis with good results.

Dr. Marks reported at the previous meeting a case of aneurism of the subclavican artery then under treatment, and for which he had amputated at the shoulder joint. The patient recently died, and he presented a full written report of the case, with a statement of the appearance of the tumor after death.

Dr. McKennan, of Sauk City, presented a written report of a case of shoulder presentation, when the uterus was ruptured during the act of version. The child was immediately delivered and the mother recovered.

He also presented the written report of another case, where the woman was very short of stature, but stout and robust, and had a deformity of the pelvis from an undue prominence of the promontory of the sacrum. Antero-posterior diameter less than three inches. She had borne twelve children, nine of which had been delivered with instruments, and were dead. The tenth, eleventh and twelfth were very small, and were born without instrumental aid. He was called to her in her thirteenth confinement, and found the pains strong and the head presenting and pressing strongly against and partially engaged in the brim of the pelvis, and so it remained for twelve hours. The doctor then turned the child and delivered by the feet, considerable force being required to extricate the head. The child was alive, and weighed some ounces over thirteen pounds.

The report of this case gave rise to some discussion as to the practicability of turning in such cases, some apprehending that in most cases the head could not be extricated.

Dr. Dalton, of Mineral Point, made an extended report of a case of aneurism of the axillary artery, which terminated in death. The patient presented himself with a large pulsating tumor in the left axilla, a small part presenting above and within the acromial articulation of the clavicle, the scapula considerably elevated, a pain at times in the left arm, with considerable swelling, and at all times a numbness, or as he expressed it, "a dead feeling extending to the ends of his fingers." He goes on to give a history of the case from the first appearance of the tumor. Told the patient the only remedy was ligation, and that the result would probably be fatal under any method of treatment. He decided to have the operation performed, and Dr. Dalton tied the left subclavican artery at the outer margin of the external scalenas, following the form of incision introduced by J. Kearney Rodgers. The operation was followed by sensible diminution in the size of the tumor and coldness of the whole surface of the arm. Case did well up to the twenty-fourth day, when the ligatures came away followed by slight hæmorrhage. Incision had entirely healed, except that portion about the ligatures, warmth nearly restored, and tumefaction almost disappeared. The case went on until the incision entirely healed, and in six days he was discharged as cured.

The operation was performed in November, and on the 9th day of March following he felt pain in the back and right shoulder, and on the night of the 10th he suddenly expired. Dr. Dalton closes his report, which is given in detail, as follows:

"I requested a *post mortem*, but could not prevail upon them to grant it. Therefore of what he died I am wholly ignorant, but from their description came to the conclusion that it might have been angina pectoris. They stated he had been suffering as usual with the pain, when he suddenly threw his right hand to his left side, screamed with pain, turning himself partially over he became easier, but his ease was of short duration. In from three to five minutes he acted in the same manner and expired.

"Whether this could in any way be connected with the aneurism, or operation, is a question I leave to the profession to decide."

Dr. Ferrin, of the committee on practical medicine, made a verbal report of an anomalous case of pain and irritability of the urethra.

The subject of puerperal convulsions having been incidentally brought up, a very general and animated discussion upon the pathology and treatment followed. President Van Dusen left the chair and participated in his usual pointed and energetic style. He was full of the subject. Being an elderly gentleman of large experience and close powers of observation, he was considered good authority upon this subject. His opinion was, that it usually occurred in persons of full or plethoric habit, and bleeding was the remedy. Such had been his practice, and he had never lost a case. He had but little faith in anæsthetics without bleeding, and such appeared to be the general opinion.

President Chadbourne, of the State University, appeared and was introduced by President Van Dusen. He gave some practical remarks upon the duties and requirements of the medical profession. He introduced the subject of establishing a medical school within the State under the law creating the university, and said, as one of the members of the committee appointed by the Regents to take into consideration the practicability of a school to be established at Milwaukee or elsewhere, he felt desirous to advance the project, but he felt that the initiative should be taken by the State Medical Society, as it must in a great measure depend upon it for its support and direction, and he hoped that the society would take action upon this when it seems to be desirable to organize such a school.

The subject of treatment of rheumatism was under discussion for sometime, and the majority seemed to favor alkalies as the most efficacious remedies.

On motion of Dr. Marks, Dr. Taggart was requested to furnish a written report of a case of spontaneous rupture of the *sphincter ani*, also that Dr. McKennan be requested to furnish a written report of his treatment of fracture of the malar bone, and Dr. Cody of a case of emphysema.

After appointment of committees and ordinary business, the society adjourned to meet on the 3d Wednesday of June next, at Madison.

